# Supplementary material for: Targeting Myeloperoxidase Activity and Neutrophil ROS Production to Modulate Redox Process: Effect of Ellagic Acid and Analogues
Source: Molecules. 2023 Jun 2;28(11):4516. doi: 10.3390/molecules28114516 (PMC10254444; doi:10.3390/molecules28114516)
Supplement: Supplementary file 1 [file molecules-28-04516-s001.zip › molecules-2371720-supplementary.pdf]

Fig. S1A

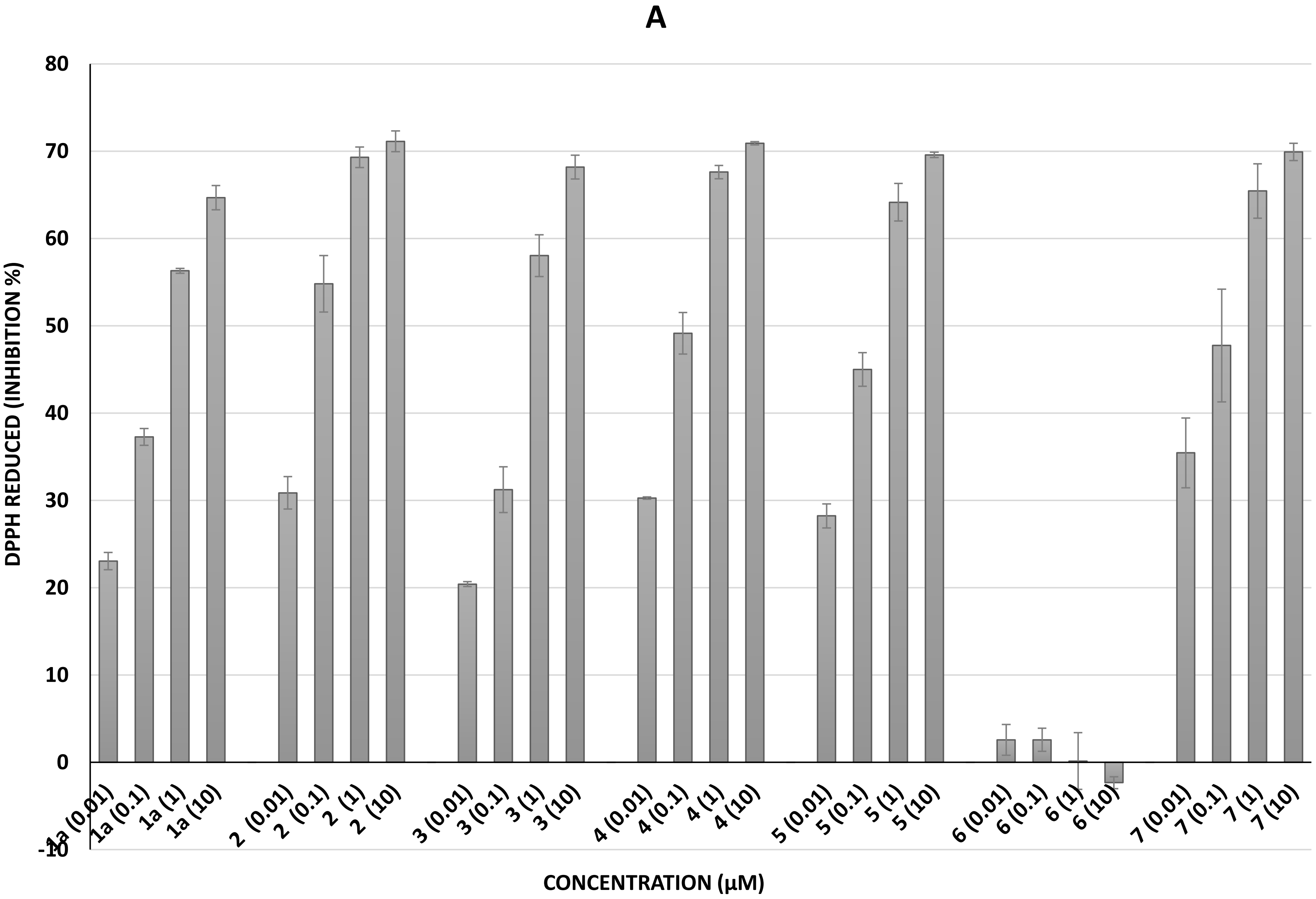
A
80
70
60
50
40
30
20
10
0
-10
DPPH REDUCED (INHIBITION %)
1a (0.01)
1a (0.1)
1a (1)
1a (10)
2 (0.01)
2 (0.1)
2 (1)
2 (10)
3 (0.01)
3 (0.1)
3 (1)
3 (10)
4 (0.01)
4 (0.1)
4 (1)
4 (10)
5 (0.01)
5 (0.1)
5 (1)
5 (10)
6 (0.01)
6 (0.1)
6 (1)
6 (10)
7 (0.01)
7 (0.1)
7 (1)
7 (10)
CONCENTRATION (μM)

Fig. S1B

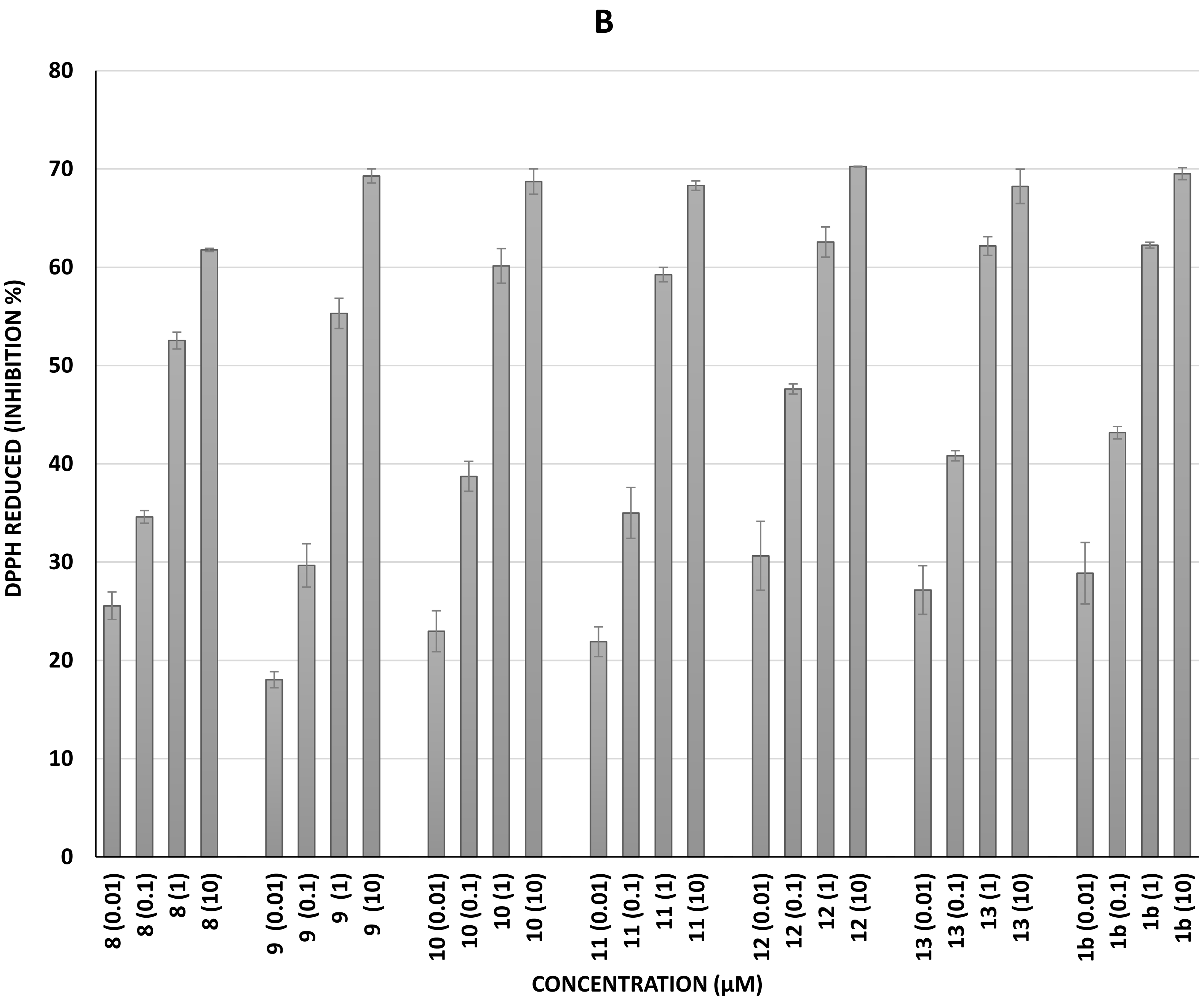
B
DPPH REDUCED (INHIBITION %)
0
10
20
30
40
50
60
70
80
8 (0.01)
8 (0.1)
8 (1)
8 (10)
9 (0.01)
9 (0.1)
9 (1)
9 (10)
10 (0.01)
10 (0.1)
10 (1)
10 (10)
11 (0.01)
11 (0.1)
11 (1)
11 (10)
12 (0.01)
12 (0.1)
12 (1)
12 (10)
13 (0.01)
13 (0.1)
13 (1)
13 (10)
1b (0.01)
1b (0.1)
1b (1)
1b (10)
CONCENTRATION (µM)

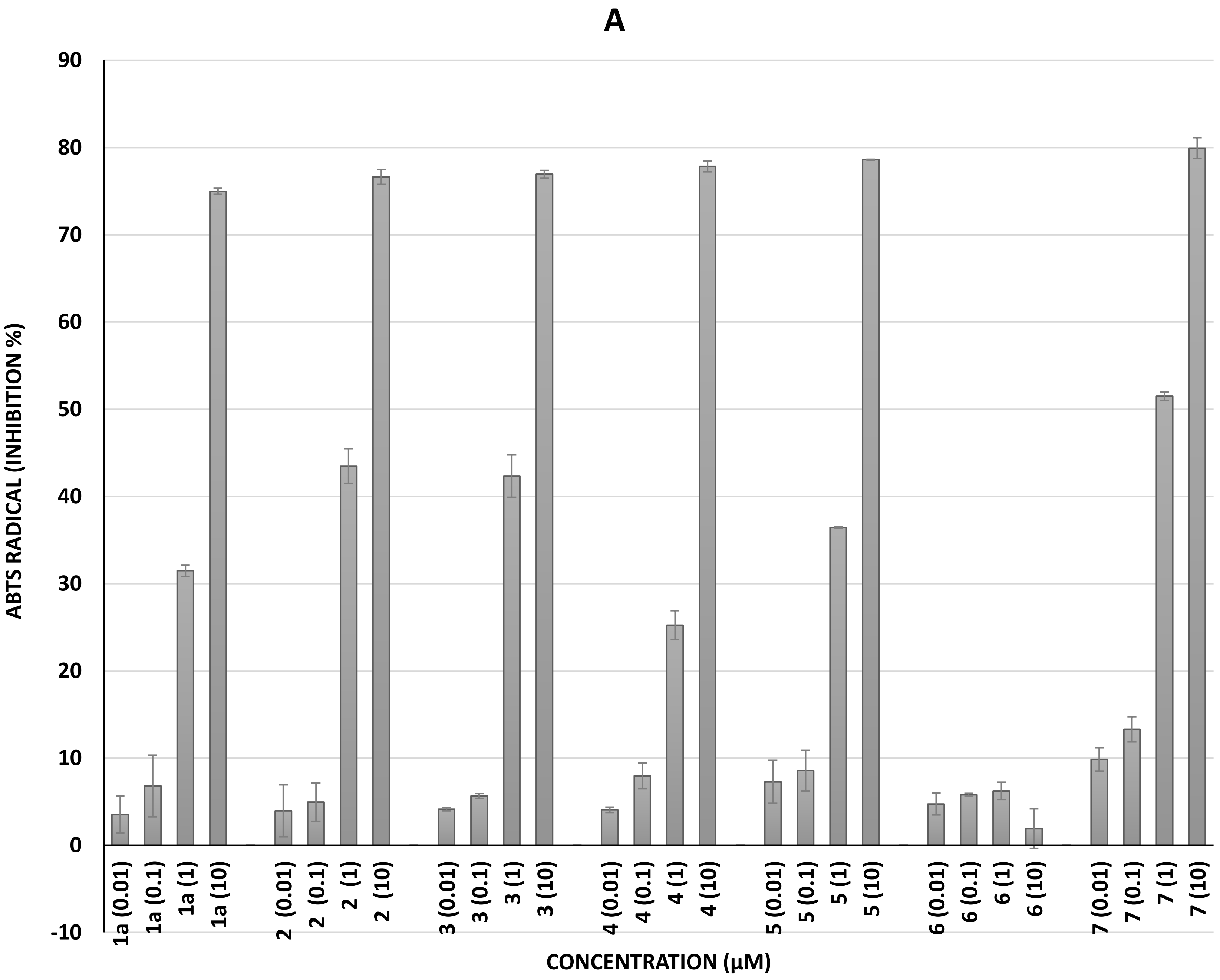
A
90
80
70
60
50
40
30
20
10
0
-10
ABTS RADICAL (INHIBITION %)
1a (0.01)
1a (0.1)
1a (1)
1a (10)
2 (0.01)
2 (0.1)
2 (1)
2 (10)
3 (0.01)
3 (0.1)
3 (1)
3 (10)
4 (0.01)
4 (0.1)
4 (1)
4 (10)
5 (0.01)
5 (0.1)
5 (1)
5 (10)
6 (0.01)
6 (0.1)
6 (1)
6 (10)
7 (0.01)
7 (0.1)
7 (1)
7 (10)
CONCENTRATION (μM)

Fig. S2B

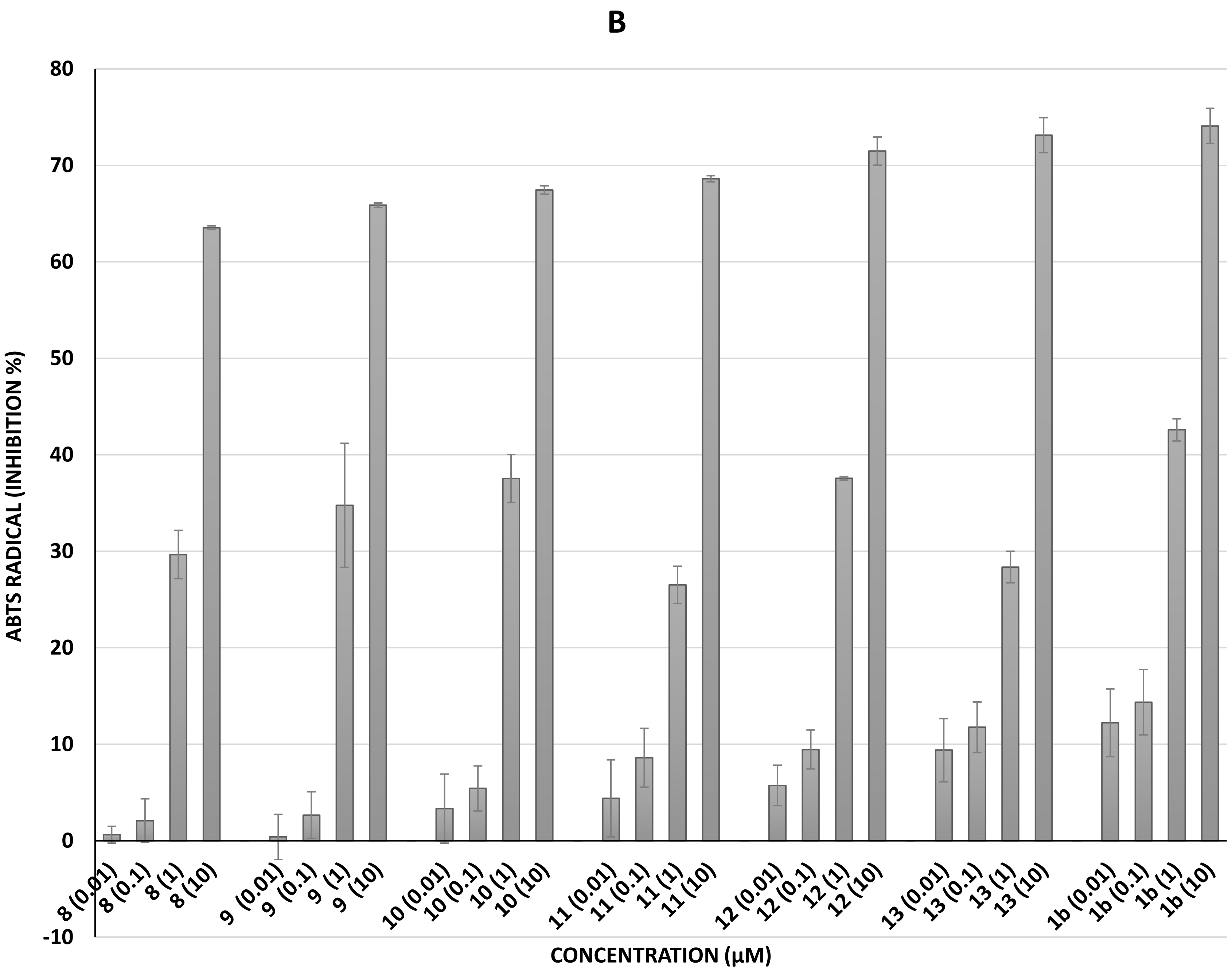
B
ABTS RADICAL (INHIBITION %)
80
70
60
50
40
30
20
10
0
-10
8 (0.01)
8 (0.1)
8 (1)
8 (10)
9 (0.01)
9 (0.1)
9 (1)
9 (10)
10 (0.01)
10 (0.1)
10 (1)
10 (10)
11 (0.01)
11 (0.1)
11 (1)
11 (10)
12 (0.01)
12 (0.1)
12 (1)
12 (10)
13 (0.01)
13 (0.1)
13 (1)
13 (10)
1b (0.01)
1b (0.1)
1b (1)
1b (10)
CONCENTRATION (μM)

Fig. S3A

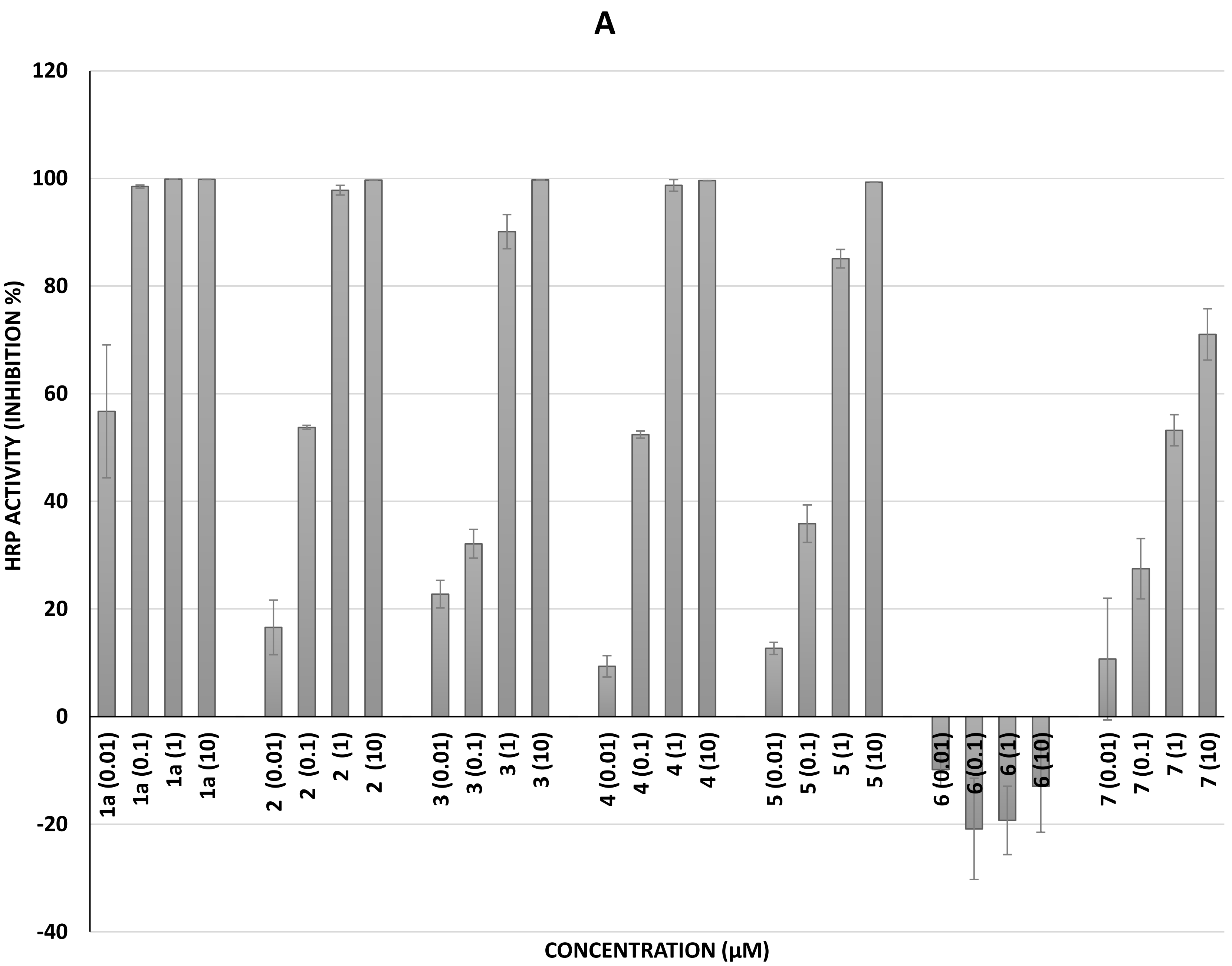
A
HRP ACTIVITY (INHIBITION %)
120
100
80
60
40
20
0
-20
-40
1a (0.01)
1a (0.1)
1a (1)
1a (10)
2 (0.01)
2 (0.1)
2 (1)
2 (10)
3 (0.01)
3 (0.1)
3 (1)
3 (10)
4 (0.01)
4 (0.1)
4 (1)
4 (10)
5 (0.01)
5 (0.1)
5 (1)
5 (10)
6 (0.01)
6 (0.1)
6 (1)
6 (10)
7 (0.01)
7 (0.1)
7 (1)
7 (10)
CONCENTRATION (μM)

Fig. S3B

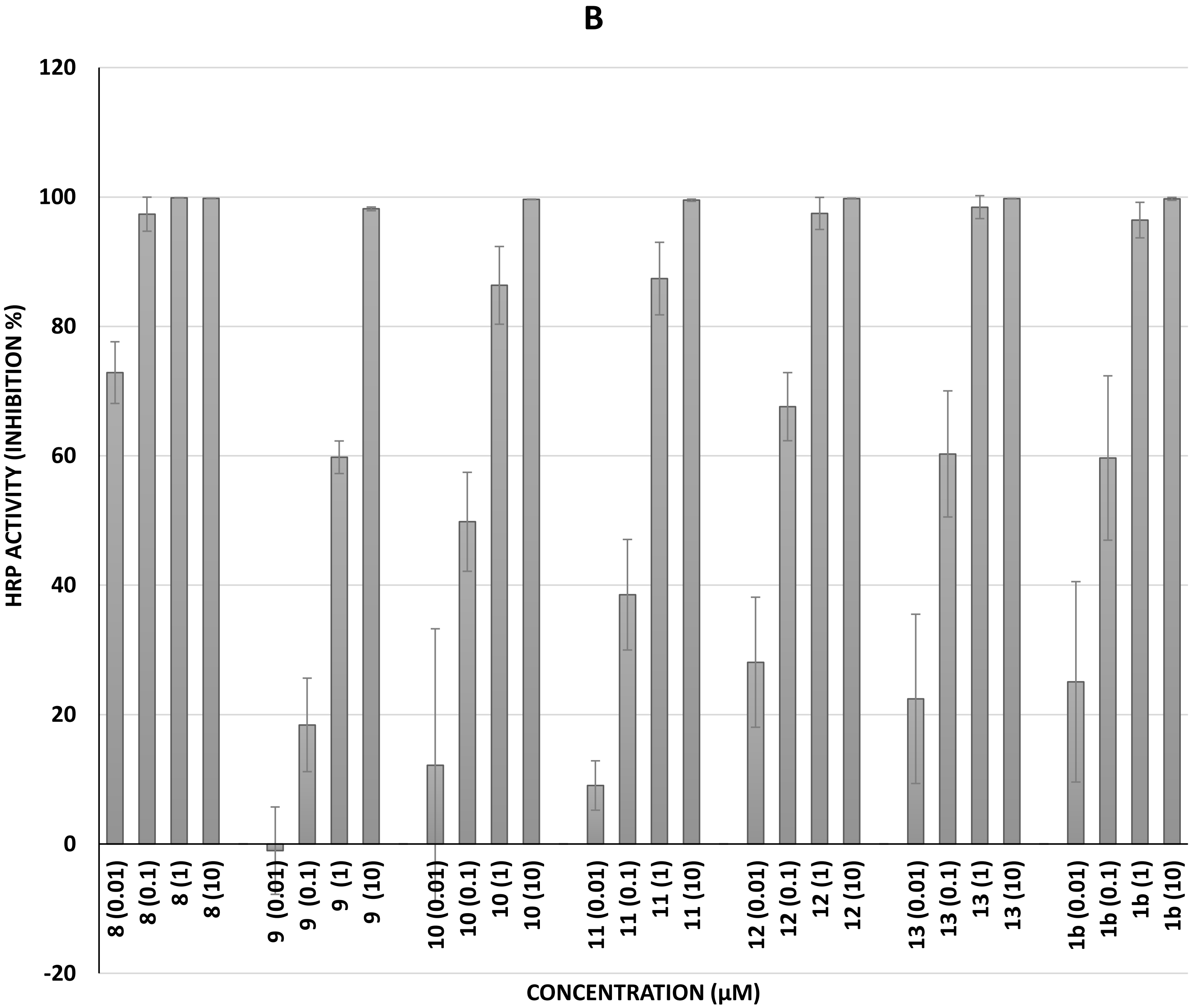
B
120
100
80
60
40
20
0
-20
HRP ACTIVITY (INHIBITION %)
8 (0.01)
8 (0.1)
8 (1)
8 (10)
9 (0.01)
9 (0.1)
9 (1)
9 (10)
10 (0.01)
10 (0.1)
10 (1)
10 (10)
11 (0.01)
11 (0.1)
11 (1)
11 (10)
12 (0.01)
12 (0.1)
12 (1)
12 (10)
13 (0.01)
13 (0.1)
13 (1)
13 (10)
1b (0.01)
1b (0.1)
1b (1)
1b (10)
CONCENTRATION (µM)

Supplementary material: Synthesis of Methyl 2-ethoxy-7-hydroxybenzo[*d*][1,3]dioxole-5-carboxylate (**y = Protected methyl gallate, 11**).

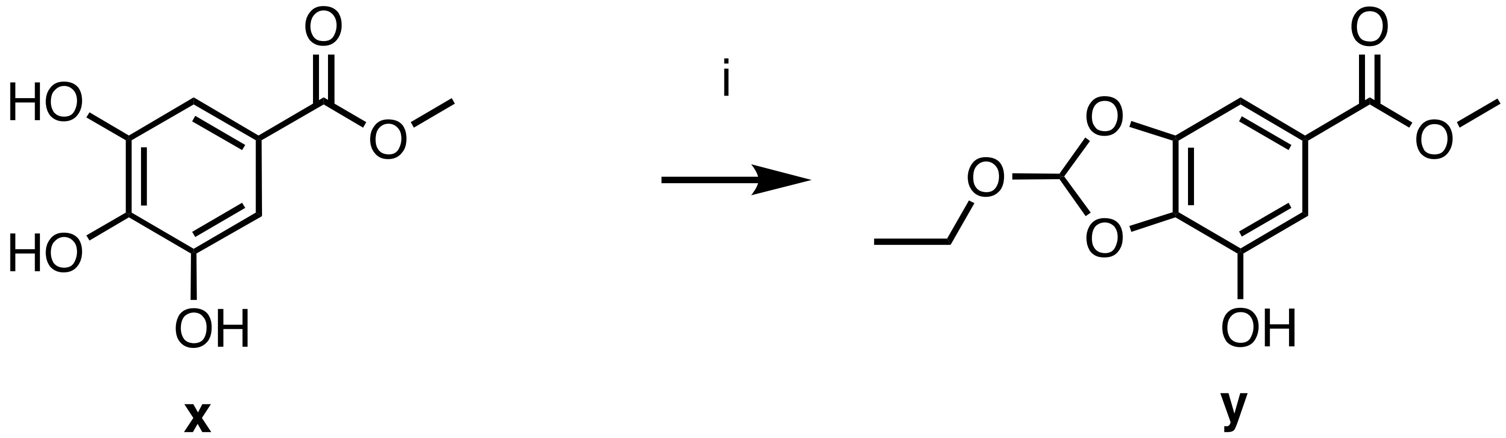

Scheme A. i: HC(OEt)3, 160°C.

A mixture of methyl 3,4,5-trihydroxybenzoate (**x**, 5,00 g, 27.2 mmol) in triethylorthoformate (5.0 ml) is heated under stirring in an open vessel at 160°C. After 4 hours, the mixture is cooled down and evaporated under vacuo. The residue is crystallysed in toluene. The obtained crystals are washed with hexane to yield **y** (3,44 g, 14.3 mmol, 52.7%). Elemental analysis: C 55.14 (55.00), H 5.05 (5.04). ^{1}H NMR (500 MHz, DMSO) δ 10.27 (s, 1H, Ar-O*H*), 7.19 (d, J = 1.6 Hz, 1H, 6-*H*)), 7.17 (s, 1H, C-*H*), 7.01 (d, J = 1.6 Hz, 1H, 4-*H*), 3.79 (s, 3H, CH_3), 3.70 (q, J = 7.1 Hz, 2H, CH_2CH_3), 1.17 (t, J = 7.1 Hz, 3H, CH_2CH_3).
